# NT5E is associated with unfavorable prognosis and regulates cell proliferation and motility in gastric cancer

**DOI:** 10.1042/BSR20190101

**Published:** 2019-05-17

**Authors:** Sifeng Hu, Fanmei Meng, Xiankun Yin, Changling Cao, Guangyong Zhang

**Affiliations:** 1 Department of General Surgery, Zhoucheng People’s Hospital, Zhoucheng 27350012, Shandong, China; 2 Department of Urology, The Affiliated Hospital of Jining Medical College, Jining 272029, Shandong, China; 3 Department of General Surgery, Qilu Hospital of Shandong University, Jinan 250000, Shandong, China

**Keywords:** biomarkers, gastric cancer, NT5E, prognosis

## Abstract

Ecto-5′-nucleotidase (NT5E) is a glycosylphosphatidylinositol anchored cell surface protein, and has been suggested to be dysregulated in most types of human cancer including gastric cancer. The aim of the present study was to present more evidence about the clinical and prognostic value of Ecto-5′-nucleotidase in gastric cancer patients, and preliminarily explore the biological function of Ecto-5′-nucleotidase in gastric cancer cells. In our study, high Ecto-5′-nucleotidase expression was observed in gastric cancer tissues and cell lines, respectively, compared with normal gastric mucosa tissues cells. Meanwhile, TCGA database also indicated that Ecto-5′-nucleotidase expression levels were notably elevated in gastric cancer tissues compared with normal gastric mucosa tissues. Furthermore, high-expression of Ecto-5′-nucleotidase was obviously associated with advanced clinical stage, deep tumor invasion, lymph node metastasis and distant metastasis in gastric cancer patients. The survival analyses of TCGA database and our study consistent suggested high Ecto-5′-nucleotidase expression was negatively correlated with overall survival time in gastric cancer patients. The univariate and multivariate Cox proportional hazards regression model showed high Ecto-5′-nucleotidase expression was an independent poor prognostic factor for gastric cancer patients. Moreover, silencing of Ecto-5′-nucleotidase expression suppressed cell proliferation, migration and invasion *in vitro* in gastric cancer. In conclusion, Ecto-5′-nucleotidase is a credible prognostic biomarker, and serves as a potential therapeutic target in gastric cancer.

## Introduction

Gastric cancer is the sixth most common cancer worldwide accounting for 1,033,701 newly diagnosed patients in 2018 [1]. Although the incidence and mortality of gastric cancer have been declining in the past decades, gastric cancer remains the second leading cause of cancer death in the world with an estimated 782,685 deaths in 2018 [1]. With the performance of clinical trials in gastric cancer, molecular targeted therapy was gradually considered as an important part of the clinical therapy such as trastuzumab [2] and apatinib [3]. Unfortunately, the prognosis of gastric cancer remains unfavorable with a 5-year overall survival of <20% [4,5]. Therefore, it is necessary to identify more novel biomarkers for guiding system management or developing effective therapeutic target in gastric cancer patients.

Ecto-5′-nucleotidase (also known as CD73) is glycosylphosphatidylinositol anchored cell surface protein, and has been suggested to be dysregulated in most types of human cancer [6,7]. In gastric cancer, Ecto-5′-nucleotidase was originally found to be methylated in primary gastric cancer tissues [8]. Subsequently, Ecto-5′-nucleotidase gene mutation was observed in gastric cancer metastasis, and predicted prognosis in gastric cancer [9,10]. Moreover, Ecto-5′-nucleotidase overexpression was found in gastric cancer tissues and serums, and associated with clinical progression in gastric cancer patients [11,12]. Due to limited sample size in previous study about the clinical significance of Ecto-5′-nucleotidase protein in gastric cancer, we present more evidence about the correlations between Ecto-5′-nucleotidase protein expression and clinicopathological characteristics including prognosis in gastric cancer patients. In addition, we tried to preliminarily explore the biological function of Ecto-5′-nucleotidase in gastric cancer cells.

## Materials and methods

### Tissue samples

The present study was approved by the Ethics Committee of The First Affiliated Hospital of Xi’an Medical University (No. 20150132), The Second Affiliated Hospital of Shaanxi University of Chinese Medicine (No. 2015-CL013) and Sheyang County People’s Hospital (No. 15043-H), and complied with the Declaration of Helsinki. All patients were aware of the present study and signed an informed consent agreement. All patients provided a written informed consent prior to the study. A total of 131 paraffin-embedded gastric cancer tissues and 45 paraffin-embedded normal gastric mucosa tissues were collected through surgery or biopsy from gastric cancer patients who were not subjected to neoadjuvant radiotherapy and chemotherapy at The First Affiliated Hospital of Xi’an Medical University, The Second Affiliated Hospital of Shaanxi University of Chinese Medicine or Sheyang County People’s Hospital. The clinicopathological characteristics such as age, gender, histological type, clinical stage, tumor depth, lymph node metastasis and distant metastasis were included in the standard questionnaire, which was used to record each gastric cancer patient in the present study.

### TCGA database

Analysis of TCGA database was performed at the GEPIA (Gene Expression Profiling Interactive Analysis, http://gepia.cancer-pku.cn/) platform. The Ecto-5′-nucleotidase expression profiles in gastric cancer samples (*n*=408) and normal gastric mucosa samples (*n*=211) were showed from TCGA database. The prognostic value of Ecto-5′-nucleotidase was assessed in gastric cancer cohort (*n*=384) from TCGA database through Kaplan–Meier method and log-rank test.

### Cell lines

Four human gastric cancer cell lines (AGS, SGC-7901, BGC-823 and NCI-N87) and human gastric mucosal epithelial cell line (GES-1) were obtained from the Cell Bank of Chinese Academy of Sciences (Shanghai, China), and cultured in Roswell Park Memorial Institute (RPMI)-1640 medium (Gibco, Rockville, MD, U.S.A.) supplemented with 10% fetal bovine serum (FBS, Gibco, Rockville, MD, U.S.A.) in a humidified incubator at 37°C with 5% CO_2_.

### Immunohistochemistry

Immunohistochemical analysis was conducted to detect Ecto-5′-nucleotidase protein expression in gastric cancer tissues and normal gastric mucosa tissues. Paraffin-embedded tissue samples were sectioned at 4-μm thick. Then, sections were deparaffinized in xylene twice for 10 min, rehydrated through graded ethanol to distilled water. After conducting antigen retrieval using a microwave for 5 min at 95°C, 3% hydrogen peroxide and 5% non-fat dried milk were respectively used to block endogenous peroxidase activity and non-specific binding activity. Subsequently, the sections were incubated with the primary Ecto-5′-nucleotidase antibody (1:100 dilution; Abcam, MA, U.S.A.) overnight at 4°C, and horseradish peroxidase-labeled anti-goat IgG secondary antibody for 30 min at room temperature. The target protein was stained with 3,3′-diaminobenzidine (DAB; Zhongshan biotech, Beijing, China) in the sections. Finally, all the sections were dehydrated and sealed after hematoxylin counterstain was completed.

The Ecto-5′-nucleotidase staining was estimated semi-quantitatively according to the combined scores of the staining intensity and percentage of positive-staining tumor cells by at least two pathologists who were blinded to the clinical data. The staining strength was graded as follows: 0 score, no coloring; 1 score, slightly yellow; 2 score, brown yellow; 3 score, tan. The percentage of positive cells was graded as follows: 0 score, positive cells <1%; 1 score, the number of positive cells ranged from 2 to 25%; 2 score, the number of positive cells ranged from 26 to 50%; 3 score, the number of positive cells ranged from 51 to 75%; 4 score, the number of positive cells >75%. Final score ≥6 and <6 were respectively defined as high-expression of Ecto-5′-nucleotidase and low-expression of Ecto-5′-nucleotidase.

### Western blot

Gastric cancer cells were lysed in RIPA buffer (Beyotime, Shanghai, China), and protein concentrations were detected by using BCA Protein Assay Kit (Beyotime, Shanghai, China). After the protein concentration of each specimen was adjusted, equal protein of each specimen was separated at sodium dodecyl sulphate-polyacrylamide gel electrophoresis (SDS-PAGE) and transferred to a polyvinylidene fluoride (PVDF) membrane (Millipore, Billerica, MA, U.S.A.). Then, the membrane was blocked by 5% non-fat dried milk for 1 h, and incubated with the primary Ecto-5′-nucleotidase antibody (1:1000 dilution; Abcam, MA, U.S.A.) overnight at 4°C. In next day, the membrane was incubated with horseradish peroxidase-conjugated secondary antibody for 2 h at room temperature. The protein bands were visualized by using the enhanced chemiluminescence Detection System (Thermo Fisher Scientific, Waltham, MA, U.S.A.).

### siRNA transfection

Small interfering RNA targeting Ecto-5′-nucleotidase (si-Ecto-5′-nucleotidase) and a scrambled negative control (si-NC) were purchased from Thermo Fisher Scientific. Gastric cancer cells were cultured 24 h, and transiently transfected with either si-Ecto-5′-nucleotidase or si-NC by using Lipofectamine® RNAiMAX Transfection Reagent (Invitrogen, Carlsbad, CA, U.S.A.) according to the manufacturer’s instructions. Knockdown efficiency was estimated by Western blot.

### Cell Counting Kit-8 (CCK-8) assay

Cell proliferation was evaluated using Cell Counting Kit-8 (CCK-8) assay kit (Dojindo, Kumamoto, Japan). Transfected gastric cancer cells (3 × 10^3^) were seeded into 96-well plates (Corning, NY, U.S.A.). About 10 μl of CCK-8 solution was then added to each well containing 100 μl of serum-free medium at 24, 48, 72 and 96 h. Then, the cells were incubated for 2 h at 37°C. Cell viability was determined in absorbance at 450 nm by a microplate reader. All assays were repeated three times.

### Transwell migration and invasion assays

Cell migration and invasion abilities were estimated by Transwell invasion and migration assays. For invasion assay, transwell chambers (8 μm pores, BD Bioscience, San Jose, CA, U.S.A.) were pre-coated with Matrigel (BD Bioscience, San Jose, CA, U.S.A.) in the upper chamber. For migration assay, there was no Matrigel in the upper chamber. Briefly, 5 × 10^4^ cells were first starved in serum-free medium, and placed in the upper chamber. Meanwhile, medium with 10% FBS added to the lower chamber. After incubating 18 h for migration and 24 h for invasion, the cells at the bottom surface of the filter membrane were stained with Giemsa solution and photographed in five random fields per insert under a light microscopy. Experiments were repeated at least three times.

### Statistical analysis

Statistical analysis was conducted by SPSS version 17.0 (Chicago, IL, U.S.A.). Comparisons between two groups were evaluated using Student’s *t*-test. The two-tailed Chi-square test or Fisher’s exact test was used to determine the significance of correlation between Ecto-5′-nucleotidase expression and clinicopathological features of gastric cancer. The overall survival curve was drawn by Kaplan–Meier method, and the significant differences of survival curves were evaluated by the log-rank test. Univariate and multivariate Cox proportional hazards regression model were used to estimate the independent predictor for overall survival of gastric cancer patients. Differences were considered to be significant at *P*<0.05.

## Results

### The expression of Ecto-5′-nucleotidase in gastric cancer

**Figure 1:**
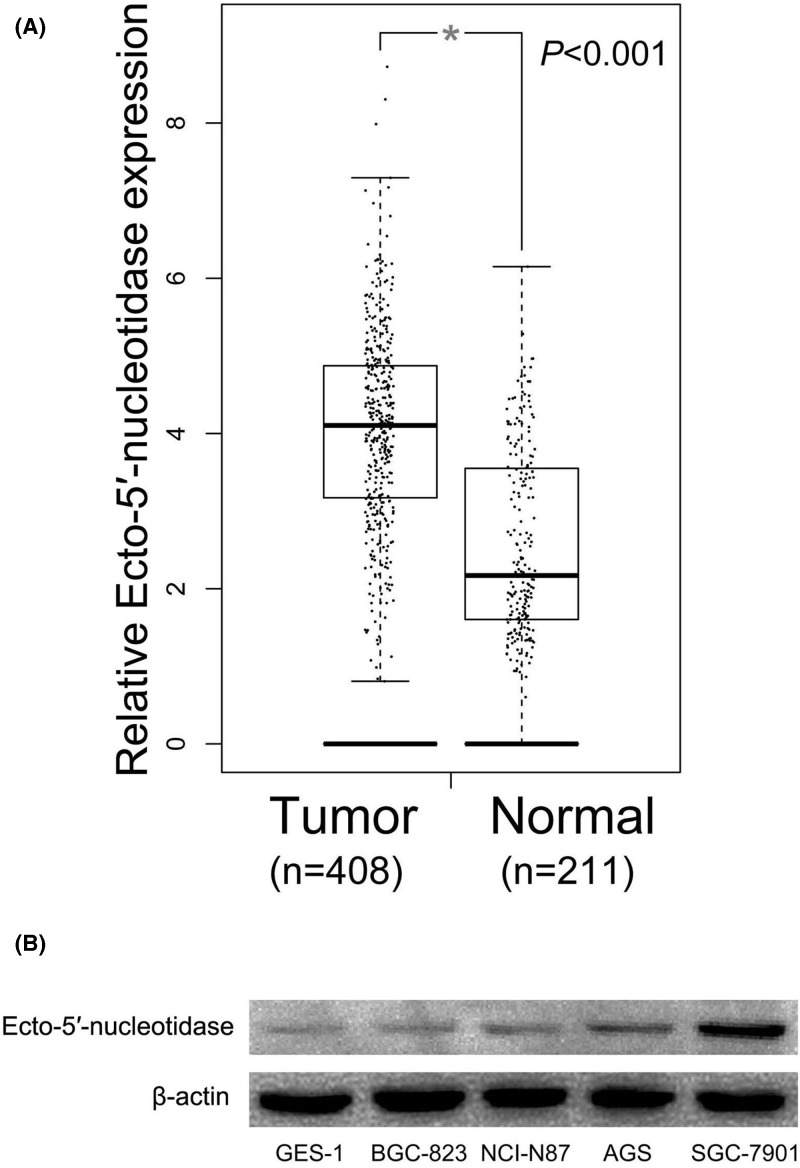
The expression of Ecto-5′-nucleotidase in gastric cancer (**A**) Ecto-5′-nucleotidase expression levels are notably elevated in gastric cancer tissues compared with normal gastric mucosa tissues. (**B**) Gastric cancer cell lines display higher level of Ecto-5′-nucleotidase expression than human gastric mucosal epithelial cell line.

**Figure 2:**
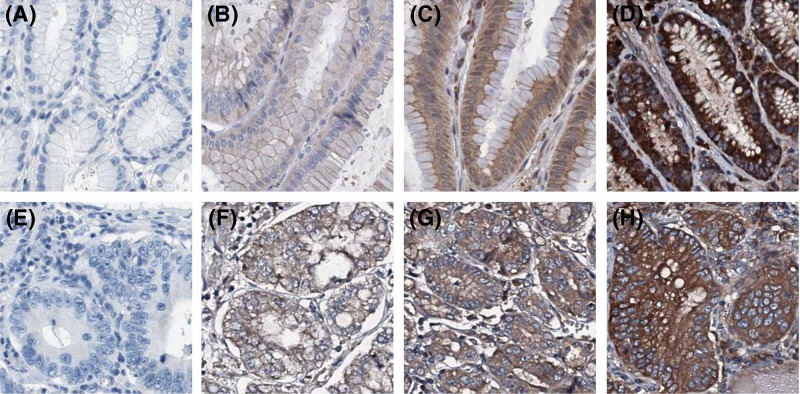
Immunohistochemical staining of Ecto-5′-nucleotidase (**A**) Negative expression of Ecto-5′-nucleotidase in normal gastric mucosa tissues. (**B**) Weak expression of Ecto-5′-nucleotidase in normal gastric mucosa tissues. (**C**) Moderate expression of Ecto-5′-nucleotidase in normal gastric mucosa tissues. (**D**) Strong expression of Ecto-5′-nucleotidase in normal gastric mucosa tissues. (**E**) Negative expression of Ecto-5′-nucleotidase in gastric cancer tissues. (**F**) Weak expression of Ecto-5′-nucleotidase in gastric cancer tissues. (**G**) Moderate expression of Ecto-5′-nucleotidase in gastric cancer tissues. (**H**) Strong expression of Ecto-5′-nucleotidase in gastric cancer tissues.

To investigate Ecto-5′-nucleotidase expression in human gastric cancer, we first observed the difference of Ecto-5′-nucleotidase expression between gastric cancer tissues and normal gastric mucosa tissues at TCGA database. In comparison with normal gastric mucosa tissues, Ecto-5′-nucleotidase expression levels were notably elevated in gastric cancer tissues (*P*<0.001, Figure 1A). To further confirm the expression of Ecto-5′-nucleotidase in gastric cancer, we performed immunohistochemical staining to detect Ecto-5′-nucleotidase protein expression in 131 gastric cancer tissues (Figure 2A–D) and 45 normal gastric mucosa tissues (Figure 2E-H). High-expression of Ecto-5′-nucleotidase was found in 72 of 131 (55.0%) gastric cancer tissues and 9 of 45 (20.0%) normal gastric mucosa tissues. Then, the statistical analysis showed Ecto-5′-nucleotidase expression definitely up-regulated in gastric cancer tissues compared with normal gastric mucosa tissues (*P*<0.001, Table 1).

**Table 1:** Ecto-5′-nucleotidase expression in between gastric cancer tissues and normal gastric mucosa tissues

Group	*n*	Ecto-5′-nucleotidase expression		***P***
		High (%)	Low (%)	
Normal	45	9(20.0)	36(80.0)	<0.001
Tumor	131	72(55.0)	59(45.0)	

The expression of Ecto-5′-nucleotidase expression was also measured in human gastric cancer cell lines (AGS, SGC-7901, BGC-823 and NCI-N87) and human gastric mucosal epithelial cell line (GES-1) through Western blot. The result of Western blot indicated that gastric cancer cell lines displayed higher level of Ecto-5′-nucleotidase expression than human gastric mucosal epithelial cell line (Figure 1B).

### Associations between Ecto-5′-nucleotidase expression and clinicopathological parameters in gastric cancer

The clinical relationship between Ecto-5′-nucleotidase expression and clinicopathological parameters of gastric cancer was further explored Chi-square test or Fisher’s exact test. The results showed that the high-expression of Ecto-5′-nucleotidase was obviously associated with advanced clinical stage (*P*<0.001, Table 2), deep tumor invasion (*P*=0.004, Table 2), lymph node metastasis (*P*=0.002, Table 2) and distant metastasis (*P*=0.007, Table 2). However, the expression of Ecto-5′-nucleotidase had no statistical association with gender, age, histological grade and HP infection.

**Table 2:** Correlations between Ecto-5′-nucleotidase expression and clinicopathological parameters in gastric cancer

Parameters	*n*	High expression (%)	Low expression (%)	***P***
Gender				
Female	48	29(60.4)	19(39.6)	0.340
Male	83	43(51.8)	40(48.2)	
Age (years)				
<50	55	29(52.7)	26(47.3)	0.662
≥50	76	43(56.6)	33(43.4)	
Histological type				
Differentiated	76	39(51.3)	37(48.7)	0.324
Undifferentiated	55	33(60.0)	22(40.0)	
Clinical stage				
I-II	59	22(37.3)	37(62.7)	<0.001
III-IV	72	50(69.4)	22(30.6)	
Tumor depth				
T1-T2	66	28(42.4)	38(57.6)	0.004
T3-T4	65	44(67.7)	21(32.3)	
Lymph node metastasis				
N0-N1	67	28(41.8)	39(58.2)	0.002
N2-N3	64	44(68.8)	20(31.3)	
Distant metastasis				
M0	119	61(51.3)	58(48.7)	0.007
M1	12	11(91.7)	1(8.3)	
HP infection				
Absent	90	48(53.3)	42(46.7)	0.579
Present	41	24(58.5)	17(41.5)	

### Relationship between Ecto-5′-nucleotidase expression and overall survival in gastric cancer

**Figure 3:**
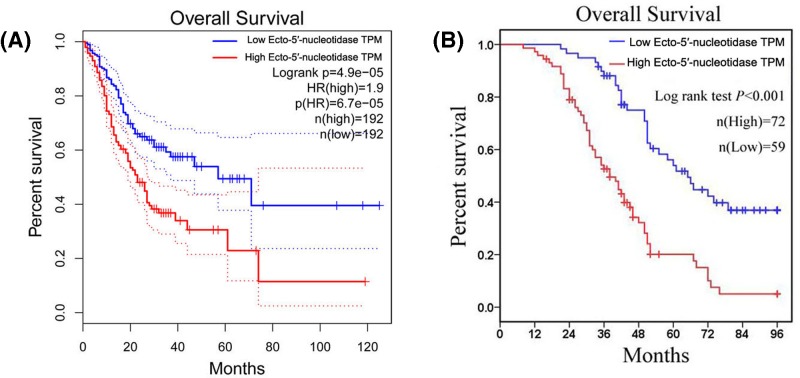
The prognostic value of Ecto-5′-nucleotidase expression in gastric cancer patients The relationship between Ecto-5′-nucleotidase expression and overall survival is evaluated at gastric cancer cohort from TCGA database (**A**) and our study (**B**) (TPM: Transcripts Per Million).

The relationship between Ecto-5′-nucleotidase expression and overall survival was primarily evaluated at gastric cancer cohort from TCGA database. We observed that high Ecto-5′-nucleotidase expression was negatively correlated with overall survival time in gastric cancer patients (*P*<0.001, Figure 3A). Then, the prognostic value of Ecto-5′-nucleotidase expression was further explored in gastric cancer patients from our study through Kaplan–Meier method and the log-rank test. The result revealed that gastric cancer patients with high-expression Ecto-5′-nucleotidase had unfavorable overall survival compared with those with low-expression of Ecto-5′-nucleotidase (*P*<0.001, Figure 3B). Furthermore, we executed univariate and multivariate Cox regression analysis for identifying the independent predictor for overall survival in gastric cancer patients. As shown in Table 3, we found clinical stage, tumor depth (*P*<0.001), lymph node metastasis (*P*<0.001), distant metastasis (*P*<0.001) and Ecto-5′-nucleotidase expression (*P*<0.001) were prognostic factors for overall survival in gastric cancer patients. Then, the result of multivariate Cox regression analysis showed high Ecto-5′-nucleotidase expression was an independent poor prognostic factor for gastric cancer patients (*P*=0.007, HR and 95%CI: 1.974 and 1.209–3.224, Table 3).

**Table 3:** Univariate and multivariate Cox regression analyses of overall survival in gastric cancer

Parameter	Univariate analysis			Multivariate analysis		
	HR	95%CI	*P*	HR	95%CI	*P*
Gender						
(Female vs. Male)	0.873	0.569–1.339	0.535			
Age						
(<50 vs. ≥50)	1.018	0.668–1.551	0.934			
Histological grade						
(Differentiated vs. Undifferentiated)	0.996	0.650–1.528	0.987			
Clinical stage						
(I-II vs. III-IV)	3.946	2.408–6.465	<0.001	2.494	0.837–7.432	0.101
Tumor depth						
(T1-T2 vs. T3-T4)	2.423	1.570–3.742	<0.001	1.863	1.167–2.975	0.009
Lymph node metastasis						
(N0-N1 vs. N2-N3)	3.535	2.193–5.698	<0.001	1.081	0.381–3.067	0.884
Distant metastasis						
(M0 vs. M1)	11.757	5.849–23.631	<0.001	5.743	2.708–12.183	<0.001
HP infection						
(Absent vs. Present)	1.099	0.704–1.714	0.679			
Ecto-5′-nucleotidase						
(Low expression vs. High expression)	3.082	1.967–4.828	<0.001	1.974	1.209–3.224	0.007

Abbreviations: 95%CI: 95% confidence interval; HR, hazard ratio.

### The biological function of Ecto-5′-nucleotidase in gastric cancer

**Figure 4:**
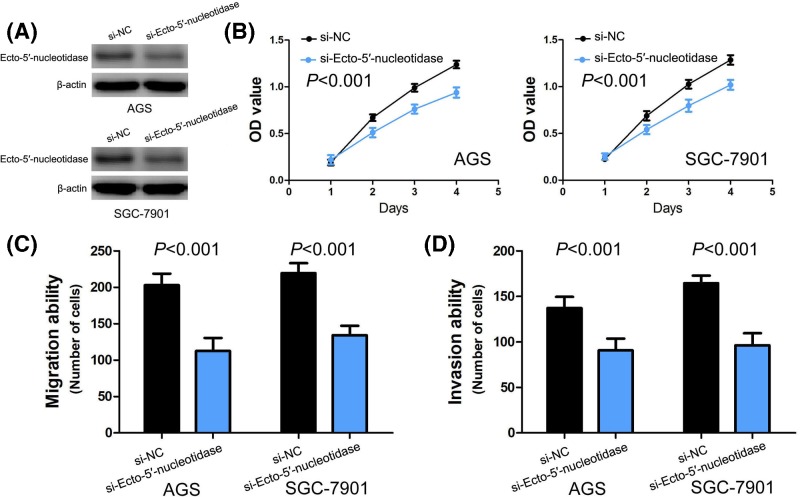
The biological function of Ecto-5′-nucleotidase in gastric cancer (**A**) The knockdown efficiency of si-Ecto-5′-nucleotidase in AGS and SGC-7901 cells is estimated by Western blot. (**B**) The effect of Ecto-5′-nucleotidase on cell proliferation is evaluated in AGS and SGC-7901 cells by CCK-8 assay (*P*<0.001 vs. si-NC). (**C**) The impact of Ecto-5′-nucleotidase on cell migration is assessed in AGS and SGC-7901 cells by transwell migration assay (*P*<0.001 vs. si-NC). (**D**) The effect of Ecto-5′-nucleotidase on cell invasion is estimated in AGS and SGC-7901 cells by transwell invasion assay (*P*<0.001 vs. si-NC).

To identify the effect of Ecto-5′-nucleotidase on gastric cancer cell proliferation, migration and invasion *in vitro*, we conducted loss-of-function in gastric cancer cells. Based on the expression of Ecto-5′-nucleotidase in four human gastric cancer cell lines (AGS, SGC-7901, BGC-823 and NCI-N87), AGS and SGC-7901 cells were chosen for following loss-of-function studies, and the knockdown efficiency of si-Ecto-5′-nucleotidase in AGS and SGC-7901 cells was estimated by Western blot (Figure 4A). We performed CCK-8 assay to assess the effect of Ecto-5′-nucleotidase on gastric cancer cell proliferation, and found that silencing of Ecto-5′-nucleotidase expression dramatically inhibited cell proliferation in AGS and SGC-7901 cells (*P*<0.001, Figure 4B). Then, the effect of Ecto-5′-nucleotidase on gastric cancer cell migration and invasion was estimated by transwell migration and invasion assays. We found silencing of Ecto-5′-nucleotidase expression also profoundly suppressed cell migration and invasion in AGS and SGC-7901 cells (*P*<0.001, Figure 4C–D).

## Discussion

Ecto-5′-nucleotidase is a glycosyl-phosphatidylinositol anchored membrane glycoprotein [13]. Originally, Ecto-5′-nucleotidase had been found to strengthen endothelial barrier function and depress leukokinesis, and be markedly affected by hypoxia and inflammatory mediators [14,15]. Subsequently, more and more human cancers have been suggested to display high Ecto-5′-nucleotidase expression, such as lung cancer [16,17], breast cancer [18], colorectal cancer [19,20], pancreatic cancer [21], gallbladder cancer [22], prostate cancer [23,24], thyroid cancer [25], and head and neck cancer [26]. However, there was discrepancy in endometrial carcinoma. Aliagas et al. [27] reported that high levels of Ecto-5′-nucleotidaseexpression were observed endometrial carcinoma samples compared with their non-pathological endometrial counterparts. On the contrary, Bowser et al. [28] showed Ecto-5′-nucleotidase expression levels were remarkably low and poorly differentiated in endometrial carcinoma samples compared with normal endometrium samples. In gastric cancer, high-expression of Ecto-5′-nucleotidase was observed in gastric cancer tissues and serums compared with normal gastricmucosal tissues and serum of healthy individuals, respectively [11,12]. In our research, we present more evidence about Ecto-5′-nucleotidase expression in gastric cancer. On the one hand, the result of TCGA database indicated that Ecto-5′-nucleotidase expression levels were notably elevated in gastric cancer tissues compared with normal gastric mucosa tissues. On the other hand, high Ecto-5′-nucleotidase expression was observed in gastric cancer tissues and cell lines through immunohistochemistry and Western blot. Furthermore, we explored the clinical relationship between Ecto-5′-nucleotidase expression and clinicopathological parameters of gastric cancer, and found high expression of Ecto-5′-nucleotidase was obviously associated with advanced clinical stage, deep tumor invasion, lymph node metastasis and distant metastasis in gastric cancer patients. Similarly, Lu et al. [12] also suggested high Ecto-5′-nucleotidase was associated with unfavorable differentiation, deep tumor invasion, more metastatic lymph nodes, distant metastasis and advanced clinical stage in gastric cancer patients. Moreover, Inoue et al. [16] showed Ecto-5′-nucleotidase overexpression was correlated with gender, smoking and histological classification in non-small cell lung cancer patients. In breast cancer patients, Yu et al. [18] showed Ecto-5′-nucleotidase overexpression was correlated with high tumor grades and present lymph node metastasis. Meanwhile, Turcotte et al. [29] found that Ecto-5′-nucleotidase overexpression predicted trastuzumab resistance in breast cancer patients. Similarly, Cushman et al. [30] also found Gene expression of Ecto-5′-nucleotidase was identified as potential biomarker for predicting cetuximab sensitivity in colorectal cancer patients. In prostate cancer patients, Yang et al. [23] suggested patients with lymph node metastasis had higher levels of Ecto-5′-nucleotidase expression than patients without lymph node metastasis. Besides, Bertonia et al. [25] showed Ecto-5′-nucleotidase overexpression was associated with large tumor size, more metastatic lymph nodes and high risk of recurrence in patients with papillary thyroid carcinoma.

The prognostic value of Ecto-5′-nucleotidase expression was estimated in many kinds of human tumor including gastric cancer [11,12], lung cancer [16,17], gallbladder cancer [22], prostate cancer [23], head and neck cancer [26,31,32,33], breast cancer [34,35], colorectal cancer [36], melanoma [37], ovarian cancer [38] and so on. Interestingly, Wang Rong et al. [39] and Jiang Tao et al. [40] respectively performed meta-analysis to estimate the prognostic value of Ecto-5′-nucleotidase expression in human cancers, and consistently found high Ecto-5′-nucleotidase expression was an efficient biomarker predicting unfavorable prognosis in human cancers. In acute lymphoblastic leukemia patients, high expression of Ecto-5′-nucleotidase was associated with unfavorable prognosis in earlier small studies [41,42]. However, high level of Ecto-5′-nucleotidase expression was found no effect on the prognosis of patients with acute lymphoblastic leukemia in larger and prospective study [43]. Due to limited sample size in previous study, we further present evidence about the association between Ecto-5′-nucleotidase expression and overall survival in gastric cancer. In our study, we found high Ecto-5′-nucleotidase expression was negatively correlated with overall survival time in gastric cancer patients both from TCGA database and our study. Meanwhile, high Ecto-5′-nucleotidase expression was identified as an independent poor prognostic factor for gastric cancer patients through univariate and multivariate Cox proportional hazards regression model. Therefore, high Ecto-5′-nucleotidase expression is credible biomarker for predicting poor clinical outcome in gastric cancer patients.

The biological function of Ecto-5′-nucleotidase in gastric cancer has not been reported. In our study, we preliminarily explored the effect of Ecto-5′-nucleotidase on gastric cancer cell proliferation, migration and invasion. We found silencing of Ecto-5′-nucleotidase expression profoundly suppressed cell proliferation, migration and invasion in gastric cancer. Similarly, Wu Ruimin et al. showed Ecto-5′-nucleotidase enhanced cell proliferation *in vitro* and tumor growth *in vivo* in colorectal cancer [20]. Moreover, Yu Jiangang suggested up-regulation of Ecto-5′-nucleotidase expression accelerated breast cancer cell proliferation [18]. Besides, Ren Zhen-Hu et al. found down-regulation of Ecto-5′-nucleotidaseexpression inhibited cell invasion, migration and epithelial-mesenchymal transition process in head and neck cancer [26].

The limit of our research was lack of the molecular mechanism of Ecto-5′-nucleotidase in gastric cancer. However, Ecto-5′-nucleotidase has been found to activate multiple oncogenic signaling pathway. In breast cancer, Ecto-5′-nucleotidase overexpression enhanced AKT/GSK-3β/β-catenin/cyclinD1 signaling pathway [18]. Moreover, inhibition of Ecto-5′-nucleotidase could depress epithelial-mesenchymal transition process and AKT/GSK-3β signal pathway in renal cell cancer [44]. Besides, Zhu Jianjie et al reported Ecto-5′-nucleotidase is a target of miR-30a-5p, and positively regulated EGFR signaling in non-small cell lung cancer [17]. Similarly, Ren Zhen-Hu et al also found Ecto-5′-nucleotidase stimulated adenosine A3R, and activate of EGF/EGFR signaling in head and neck squamous cell carcinoma [26]. In future research, we will try to investigate the molecular mechanism of Ecto-5′-nucleotidase in gastric cancer.

In conclusion, Ecto-5′-nucleotidase expression is increased in gastric cancer tissues and cells, and associated with advanced clinical stage, deep tumor invasion, lymph node metastasis and distant metastasis in gastric cancer patients. Silencing of Ecto-5′-nucleotidase expression suppresses cell proliferation, migration and invasion in gastric cancer.

## Abbreviations

CCK-8: Cell Counting Kit-8
